# Then there were five: a reexamination of the ant genus *Paratrechina* (Hymenoptera, Formicidae)

**DOI:** 10.3897/zookeys.422.7779

**Published:** 2014-06-30

**Authors:** John S. LaPolla, Brian L. Fisher

**Affiliations:** 1 Department of Biological Sciences, Towson University, Towson, Maryland 21252 USA; 2 Department of Entomology, California Academy of Sciences, Golden Gate Park, 55 Music Concourse Drive, San Francisco, CA 94118 USA

**Keywords:** Afrotropics, Madagascar, new species, *Paratrechina*, *Prenolepis*, taxonomy

## Abstract

The ant genus *Paratrechina* is reexamined based on the discovery of two new species from Madagascar (*P. ankarana* **sp. n.** and *P. antsingy* **sp. n.**). *Paratrechina kohli*, a species known from central Africa, is transferred to *Paratrechina* from *Prenolepis* based on a new morphological interpretation of the genus and an updated morphological diagnosis of the genus is provided. This means that other than the widespread *P. longicornis*, whose origins remain uncertain, all *Paratrechina* are restricted either to the Afrotropical or Malagasy regions. It would also appear that of the five *Paratrechina* species now known, three are from dry forest habitats. With this reexamination of the genus, the possible origins of *P. longicornis* are discussed. A key to the genera of the *Prenolepis* genus-group is provided, as is a key to the workers of *Paratrechina*. In addition, we also designate a lectotype for *Paratrechina kohli*.

## Introduction

During the course of revising the ant genera *Paraparatrechina* (LaPolla et al. 2010c) and *Nylanderia* (LaPolla and Fisher, in prep) in Madagascar, two species were discovered that were initially thought to belong to the genus *Prenolepis*. This was particularly interesting because the genus is unknown from the Malagasy region. Despite recent definition changes to *Prenolepis*, following the removal of the Neotropical species to their own genus, *Zatania* (LaPolla et al. 2012), the genus remains poorly understood taxonomically. There were two features of these two new species from Madagascar that gave us pause as to their potential placement in *Prenolepis*: they did not possess the characteristic mesonotal constriction immediately behind the pronotum that is present in most *Prenolepis*, and the eyes were towards the midline of the head, whereas most *Prenolepis* have high eyes relative to the midline of the head (LaPolla et al. 2010a). Given that these same characters are variable within *Zatania*, it was assumed *Prenolepis* was displaying a similar level of variability. However, to be certain, the DNA of one of the new Malagasy species was sequenced (due to sample’s old age this was impossible for the second species) and compared against a large dataset of other formicine ants (Ward, unpublished data). The molecular data strongly suggested that the species from Madagascar was not a *Prenolepis* but rather belonged in *Paratrechina*. This in retrospect makes morphological sense because it renders the mesosomal constriction coupled with high eyes and long palps an unequivocal diagnostic feature of *Prenolepis*. It also provides for a more complete understanding of morphological variation observed within *Paratrechina*.

Here we describe those two species in the genus *Paratrechina* and make some definition changes to the genus, including moving a species from *Prenolepis* to *Paratrechina*, based on the discovery of these new species. We also provide an updated key to the genera of the *Prenolepis* genus-group.

## Materials and methods

Specimens examined for this study are deposited in the following institutions:

BMNH Natural History Museum, London, U.K.

CASC California Academy of Sciences, San Francisco, USA

PBZT Parc Botanique et Zoologique de Tsimbazaza, Antananarivo, Madagascar

USNM National Museum of Natural History, Washington, DC, USA

All measurements were taken at 80× power with a Leica M125 microscope using an orthogonal pair of micrometers, recorded to the nearest 0.001 mm, and rounded to two decimal places for presentation. Multiple specimens were measured for each species, and minimum and maximum measurements and indices are presented. All measurements are given in millimeters. Digital color images were created using a Leica DFC425 digital camera. Leica Application Suite software (ver. 3.8) was used for images. Each imaged specimen is uniquely identified with a specimen-level unique identifier (e.g. CASENT0454372). Morphological terminology for measurements and indices employed throughout are defined as (following LaPolla et al. 2011a, LaPolla et al. 2011b):

EL (Eye Length): maximum length of compound eye in full-face view.

GL (Gaster Length): the length of the gaster in lateral view from the anteriormost point of the first gastral segment (third abdominal segment) to the posteriormost point.

HL (Head Length): the length of the head proper, excluding the mandibles; measured in full-face view from the midpoint of the anterior clypeal margin to a line drawn across the posterior margin from its highest points.

HW (Head Width): the maximum width of the head in full-face view.

MMC (Mesonotal Macrosetal Count): the number of erect macrosetae on mesonotum to one side of sagittal plane.

PW (Pronotal Width): the maximum width of the pronotum in dorsal view.

PFL (Profemur Length): the length of the profemur from its margin with the trochanter proximally to its margin with the tibia distally.

PMC (Pronotal Macrosetal Count): the number of erect macrosetae on pronotum to one side of sagittal plane.

SL (Scape Length): the maximum length of the antennal scape excluding the condylar bulb.

SMC (Scape Macrosetal Count): the number of erect macrosetae on the scape visible in full frontal view. This count does not include the terminal cluster of setae often found around the joint of the scape and the funiculus.

TL (Total Length): HL+WL+GL

WL (Weber’s Length): in lateral view, the distance from the posteriormost border of the metapleural lobe to the anteriormost border of the pronotum, excluding the neck.

CI (Cephalic Index): (HW/HL) × 100

REL (Relative Eye Length Index): (EL/HL) × 100

REL2 (EL/HW) × 100

SI (Scape Index): (SL/HW) × 100

## Results and discussion

LaPolla et al. (2013) provided a diagnosis for *Paratrechina*, which was based on the only two species known from the genus at that time: *Paratrechina longicornis* (Latreille, 1802) and *Paratrechina zanjensis* LaPolla, Hawkes & Fisher, 2013. Based on both morphological similarity and molecular data (Ward, unpublished data), it would appear that those two species are sister taxa, so the earlier diagnosis provided for the genus was morphologically restrictive compared to what we now know is a morphologically more diverse genus. This emphasizes that there is a need to keep documenting new species in this genus-group, in particular in some of the smaller genera (i.e. *Euprenolepis*, *Zatania*) where new species might cause some additional morphological definition changes. In more speciose genera, such as *Nylanderia* and *Paraparatrechina*, this appears to be less of a concern, as the morphological definitions provided (LaPolla et al. 2010a) have not needed to be amended despite the recent discovery of dozens of new species in each genus (Kallal and LaPolla 2012; LaPolla et al. 2010b; LaPolla et al. 2011b).

The addition of three species to *Paratrechina* not only allows for the more complete morphologically based definition provided, but it also simplifies the morphological diagnosis of *Prenolepis*. With the removal of *Prenolepis kohli* from *Prenolepis*, the mesonotal constriction immediately behind the pronotum becomes a characteristic of all *Prenolepis* species. Such a constriction is also seen in the Southeast Asian genus *Euprenolepis* (LaPolla 2009), as well as in the Caribbean and Mesoamerican genus *Zatania*, but only in three of the five known species (LaPolla et al. 2012).

With our placement of *Paratrechina kohli* into *Paratrechina* and the description of two endemic Malagasy species, the center of *Paratrechina* diversity is now squarely in the Afrotropical and Malagasy regions, which raises some interesting questions as to the origin of the now pantropical *Paratrechina longicornis* (LaPolla et al. 2013). A review of the argument that *Paratrechina longicornis* is Asian in origin is provided by Wetterer (2008) and LaPolla et al. (2013) (i.e., on the observation that *Paratrechina longicornis* has only been found in undisturbed habitats in tropical Asia). However, since *Paratrechina zanjensis* appears to be a miombo woodland specialist and its sister taxon is almost certainly *Paratrechina longicornis*, it raises the possibility that *Paratrechina longicornis* might be an African woodland specialist as well. Given the fact that most African woodland habitat has been impacted to some extent by humans, it might be difficult to prove that *Paratrechina longicornis* is in fact native there. It is worth noting that the two new Malagasy species described here are native to dry forest habitats on limestone outcrops. While certainly *Paratrechina kohli* is a rainforest species, we cannot dismiss the possibility that it is the only species native to rainforests in the genus. There is one report of *Paratrechina longicornis* from native forest in Cameroon (Dejean et al. 1996). However, only one specimen of *Paratrechina longicornis* (out of 62,708 specimens) was collected from 15 forest sites in Tanzania (P. Hawkes, pers. comm.), so the conclusions of the previous study remain equivocal. Clearly, the question of the native range of *Paratrechina longicornis* remains an open one, but with the discovery of multiple *Paratrechina* species in the Afrotropical and Malagasy regions, a more complete survey is needed, and an Asian origin for the species now seems questionable.

### Diagnosis of the genus

For only one species are all castes known (*Paratrechina longicornis*); therefore we provide only a worker-based diagnosis for the genus.

Monomorphic, medium sized (2.1–3.2 mm in total length); ranging from almost black to brownish-yellow, with lighter mandibles, antennae (especially funicular segments towards tips) and legs (especially distal portion of tibiae and tarsi). Head with medially erect macrosetae roughly paired, extending through the medial portion of clypeus. Antennae 12 segmented; scapes long, with scape index above 140, in most species around or above 200 (SI range 143–226). Scapes with a dense layer of pubescence. Head is usually distinctly longer than wide, with cephalic index below 100 (CI range 71–94); posterolateral corners rounded, with straight posterior margin. Eyes large relative to head width (REL2 greater than 25); eyes distinctly convex, extending beyond head margin in full-face view. Mandibles in all species, except *Paratrechina kohli*, with 5 teeth; in *Paratrechina kohli* 8 teeth present, with 7^th^ tooth on basal angle of mandible and 8^th^ tooth on inner mandibular margin; mandalus large and anteriorly placed; palps very long; palp formula 6:4. Mesosoma elongated, most robust in *Paratrechina kohli*; most gracile in *Paratrechina longicornis*; propodeal dorsal face variable from either nearly flat (*Paratrechina longicornis*) or distinctly convex (*Paratrechina antsingy*); propodeum without macrosetae, anteriorly occasionally with a sparse layer of pubescence; pronotal setal count 6–12 (both sides of notum); mesonotal setal count 4–8 (both sides of notum). In lateral view, petiole cuneate, broadly rounded dorsally, with much longer posterior face and not surpassing the height of the propodeum. Legs distinctly long (profemur length 0.6–1.0 mm). Gaster robust, covered in abundant erect macrosetae.

### Updated worker-based key to the genera of the *Prenolepis* genus-group

This key is modified from that found in LaPolla et al. (2012).

**Table:** 

1	Maxillary palps with five or fewer segments; species often strongly polymorphic, with a major and minor caste expressed	2
–	Maxillary palps with six segments; species monomorphic to slightly polymorphic, with no discernable major or minor caste expressed	4
2	Polymorphic Afrotropical species	*Paraparatrechina* (*weissi* species-group)
–	Monomorphic or polymorphic Australasian/Indoaustralian species	3
3	Eyes large, typically REL ≥ 20 (one exception: *Euprenolepis negrosensis*); labial palps typically with four segments; mesothorax constricted immediately behind pronotum; mandalus large and conspicuous, usually visible without dissection of mandible (This character can be difficult to determine unless the mandible is dissected. It is only required if specimens have four-segmented labial palps AND five mandibular teeth.)	*Euprenolepis*
–	Eyes small, REL < 20, typically 15 or less; labial palps typically with two or three segments; mesothorax typically not constricted immediately behind pronotum; mandalus small and inconspicuous, usually not visible without dissection of mandible	*Pseudolasius*
4	Mesothorax constricted immediately behind pronotum	5
–	Mesothorax not constricted immediately behind pronotum	6
5	Head round in general appearance, with rounded, indistinct posterolateral corners; mesothorax always constricted immediately behind pronotum, typically with distinctly convex eyes (if eyes not distinctly convex, then head is distinctly round)	*Prenolepis*
–	Head rectangular in general appearance, with more angular, distinct posterolateral corners; eyes relatively flat	*Zatania* (in part)
6	Mandibles with five or eight teeth (one species, *Paratrechina kohli*, has eight teeth, one on basal angle and one on the inner mandibular margin, all others have five teeth)	7
–	Mandibles with 6 teeth (a few species of *Nylanderia* have seven teeth, but never five or eight teeth as above)	8
7	Erect setae (one pair) present on propodeum; erect setae on head form a pattern of four setae along posterior margin and six or seven rows from posterior margin to clypeal margin; femora and tibiae lacking large erect setae	*Paraparatrechina* (in part)
–	Erect setae absent on propodeum; erect setae on head scattered across surface; femora and tibiae with large erect setae	*Paratrechina*
8	Scapes with either pubescence or very short abundant erect macrosetae (macrosetae no longer than width of scapes); profemur length greater than 0.8 mm	*Zatania* (in part)
–	Scapes usually with macrosetae that are much longer than width of scapes; in a few species no macrosetae present, but profemur length is always less than 0.8 mm	*Nylanderia*

### Synopsis of *Paratrechina* species

*Paratrechina ankarana* sp. n. Madagascar

*Paratrechina antsingy* sp. n. Madagascar

*Paratrechina kohli* (Forel, 1916), comb. rev. DR Congo

*Paratrechina longicornis* (Latreille, 1802) Pantropical tramp, origin uncertain

=*Paratrechina currens* Motschoulsky, 1863. Junior synonym of *longicornis* by Emery, 1892: 166. Neotype designated by LaPolla et al. 2010b: 1

=*Paratrechina gracilescens* (Nylander, 1856). Synonymy with *longicornis* by Roger 1863: 10

= *Paratrechina longicornis hagemanni* (Forel, 1901). Junior synonym of *longicornis* by Wheeler 1922: 942. Revived from synonymy by Emery 1925: 217. Junior synonym of *longicornis* by LaPolla et al. 2010a: 128.

= *Paratrechina vagans* (Jerdon, 1851). Junior synonym of *longicornis* by Dalla Torre 1893: 179; Forel 1894: 408

*Paratrechina zanjensis* LaPolla, Hawkes & Fisher, 2013 Angola, Mozambique and Tanzania

### Worker-based key to *Paratrechina* species

**Table:** 

1	Mandibles with eight teeth; six teeth on masticatory margin, one tooth on basal angle, another tooth on inner mandibular margin; head cuticle densely rugorecticulate	*Paratrechina kohli*
–	Mandibles with five teeth; head cuticle smooth and shiny	2
2	Scapes without macrosetae; dorsum of propodeum almost flat to very shallowly domed	*Paratrechina longicornis*
–	Scapes with macrosetae; dorsum of propodeum rounded, dome-like	3
3	Macrosetae dark brown, occasionally with lighter tips; SI greater than 195; Afrotropical region	*Paratrechina zanjensis*
–	Macrosetae yellow to whitish; SI less than 195; Malagasy region	4
4	Dark brown, with cuticle giving a faint greenish-blue reflection; propodeum with fine striations across surface	*Paratrechina ankarana*
–	Brown with patches of lighter cuticle across body, giving species a mottled appearance; propodeum smooth and very shiny	*Paratrechina antsingy*

### Species descriptions

#### Paratrechina ankarana sp. n.

Taxon classificationAnimaliaHymenopteraFormicidae

http://zoobank.org/484B4368-33CB-4BFB-B387-C6F6A0D49A0D

Figs 1–3

##### Holotype worker.

MADAGASCAR: Province Antsiranna, Rés. Spéc. Ankarana; 22.9 km 224° SW Antivorano Nord; 80 m; 12°55'S, 49°7'E; 10-16.ii.2001; Fisher et al.; CASENT0454372 (CASC). 6 paratype workers with the same locality information as the holotype (CASC, USNM); 9 paratype workers, Antsiranna, Rés. Spéc. Ankarana; 22.9 km 224° SW Antivorano Nord; 210 m; 12°52'S, 49°14'E; 16-21.ii.2001; Fisher et al. (BMNH, CASC, PBZT, USNM).

##### Worker diagnosis.

Dark brown, with cuticle giving a faint greenish-blue reflection; propodeum with fine striations across surface; SI less than 195.

Worker. *Measurements (n=6)* TL: 2.60–2.84; HW: 0.60–0.63; HL: 0.66–0.72; EL: 0.18–0.20; SL: 1.10–1.17; PW: 0.42–0.46; WL: 1.01–1.07; GL: 0.90–1.14; PFL: 0.80–0.90; CI: 85–92; REL: 27–29; REL2: 31–33; SI: 184–191; SMC: 24–30; PMC: 3–6; MMC: 3–4.

Dark brown; antennae lighter with trochanters yellow to white; cuticle smooth and shiny, except on propodeum which posseses fine striations across surface; under microscrope view cuticle has a faint greenish-blue reflection; abundant macrosetae across scapes, head, pronotum, mesonotum, and gaster; scapes with short pubescence. Head ovate; posterolateral corners rounded with complete posterior margin; midpoint of eyes at approximately midline of head; eyes convex; 3 small ocelli present; mandibles with 5 teeth; apical tooth the longest, 3^rd^ tooth from apical shortest, remainder about the same size; outer mandibular surface with slight striations across surface. In lateral view, pronotum rises in a straight margin towards mesonotum with only slight convexity towards mesonotal margin; propodeum large and bulbous, making division between dorsal and declivitous faces difficult; metanotal suture with distinct impression that extends down along the mesopleural/metapleural margin; mesopleuron with darkened ridge along impression.

**Figures 1–3.**
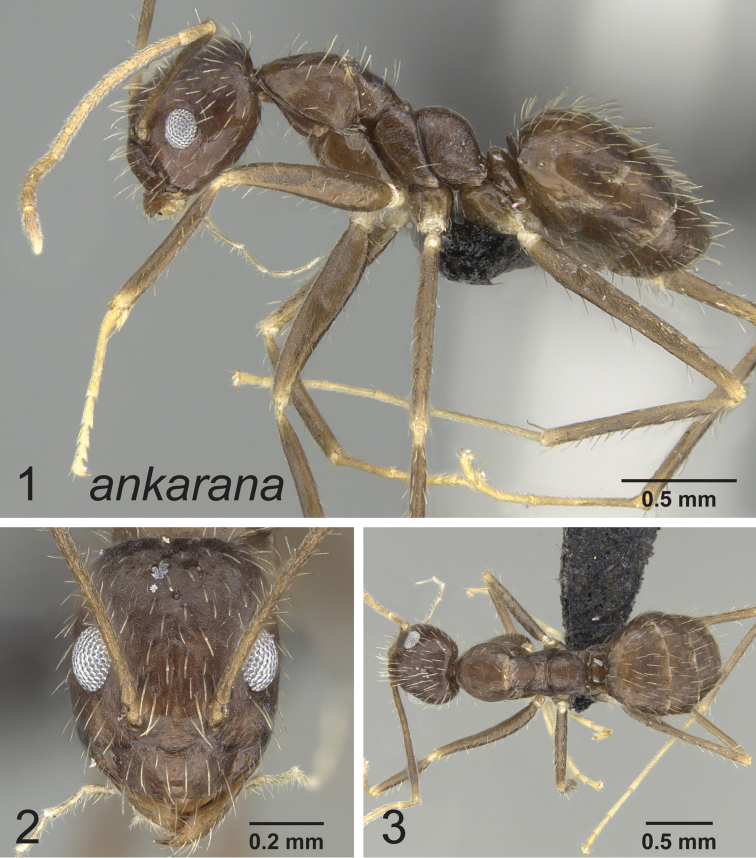
Lateral, full face and dorsal view of body. *Paratrechina ankarana* holotype worker CASENT0454372.

##### Etymology.

The specific epithet is derived from the name of the reserve where the species was found.

#### Paratrechina antsingy sp. n.

Taxon classificationAnimaliaHymenopteraFormicidae

http://zoobank.org/49E90C54-69FC-4D3B-91D0-9C5EACAFC875

Figs 4–6

##### Holotype worker.

MADAGASCAR: Province Antsiranna, Montagne Français, 7.2 km 142° SE Diego Suarezi, 180m, 12°19'S, 49°20'E, 22–28.ii.2001, Fisher et al. CASENT0906916 (CASC). 12 paratype workers with same locality information as holotype (BMNH, CASC, USNM).

##### Worker diagnosis.

Cuticle smooth and very shiny; brown with patches of lighter cuticle across body, giving species a mottled appearance; SI less than 195.

Worker. *Measurements (n=6)* TL: 2.07–2.69; HW: 0.58–0.60; HL: 0.67–0.70; EL: 0.17–0.19; SL: 1.02–1.08; PW: 0.40–0.44; WL: 0.97–1.02; GL: 0.76–1.05; PFL: 0.74–0.78 CI: 84–89; REL: 26–28; REL2: 29–32; SI: 177–180; SMC: 24–30; PMC: 4–5; MMC: 3–4.

Brown with lighter areas on mandibles, antennae, lateral portions of head, mesonotum, pronotum, mesonotum, trochanters and joints of femora; first gastral tergite with mottled lighter and darker areas; cuticle smooth and very shiny, except for mesonotum, metanotal area and propodeum being slightly rugolose; scapes, head, pronotum, mesonotum and gaster with abundant macrosetae; scapes with short, appressed pubescence. Head ovate; posterolateral corners rounded with complete posterior margin; midpoint of eyes at approximately midline of head; eyes convex; 3 small ocelli present; mandibles with 5 teeth; apical tooth the longest, 3^rd^ tooth from apical shortest, remainder about the same size; outer mandibular surface with slight striations across surface. In lateral view, pronotum convex; pronotal/mesonotal margin with mesonotum raised slightly above margin; propodeum convex with dorsal face rising steeply to a rounded peak with steep declivitous face; metanotal suture with distinct impression that extends down along the mesopleural/metapleural margin.

**Figures 4–6.**
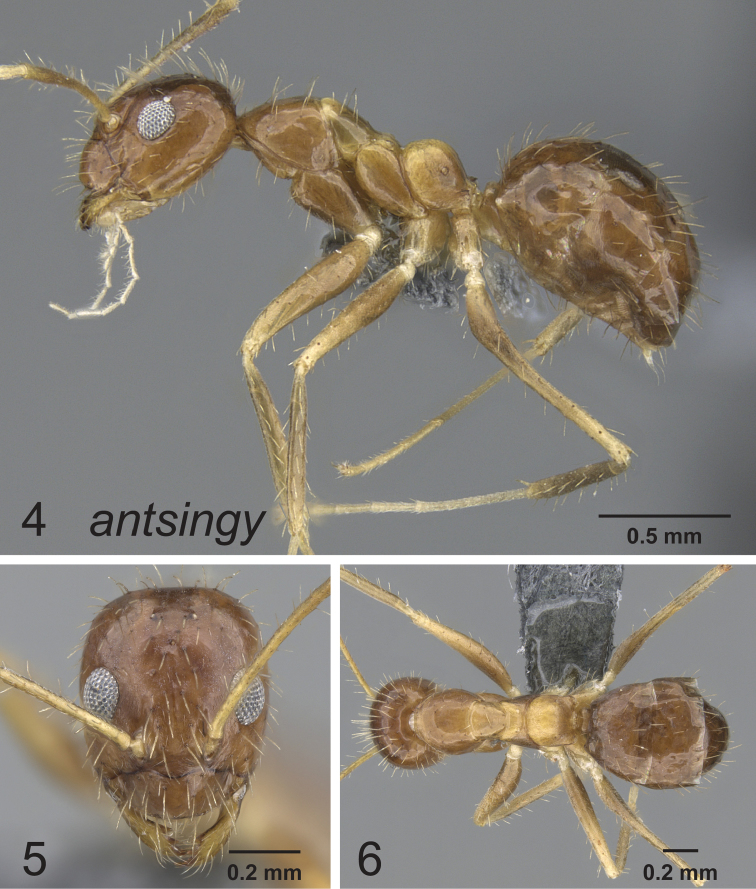
Lateral, full face and dorsal view of body. *Paratrechina antsingy* holotype worker CASENT0906916.

##### Etymology.

The specific epithet is derived from the name of the province where the species was found.

#### Paratrechina kohli

Taxon classificationAnimaliaHymenopteraFormicidae

(Forel, 1916) comb. rev.

Figs 7–9

Prenolepis kohli Forel, 1916: 438 (worker and queen decribed). Syntype worker and queen, Democratic Republic of the Congo (Kohl) (MHNG) [examined]. Combination in *Paratrechina* (*Nylanderia*): Emery 1925: 218; in *Prenolepis*: LaPolla et al. 2010a: 129. Lectotype [designated here], pinned worker, Congo (Kohl) (MHNG: CASENT0907121) [examined]; Paralectotype [designated here], pinned queen with same data as lectotype (MHNG: CASENT0907122) [examined].

##### Worker diagnosis.

Head and mesosoma light brown to yellow with slightly darker gaster; mesosoma and head densely rugorecticulate; mandible with 8 teeth, one tooth on basal margin, another on inner mandibular margin.

Worker. *Measurements (n=6)* TL: 2.9–3.0; HW: 0.78–0.90; HL: 0.93–1.03; EL: 0.21–0.25; SL: 1.22–1.43; PW: 0.54–0.6; WL: 1.3–1.61; GL: 1.12–1.37; PFL: 0.84–1.04 CI: 79–85; REL: 22–25; REL2: 27–30; SI: 143–169; SMC: 0; PMC: 3–5; MMC: 1–3.

Light brown with darker gaster; head and mesosomal cuticle densely rugorecticulate; head, pronotum, mesonotum and gaster with scattered macrosetae; scapes without macrosetae, with layer of dense pubscence. Head subrectangular; posterolateral corners with slight angles; most of eyes above midline of head; eyes convex; no ocelli apparent; mandibles with with 8 teeth, one tooth on basal margin, another on inner mandibular margin; outer mandibular surface with slight striations across surface. In lateral view, pronotal margin nearly straight, at less than 45° angle rise towards mesonotum; pronotal/mesonotal margin with mesonotum raised slightly above margin; propodeum convex with rounded dorsal face and longer, steep declivitous face; metanotal suture with distinct impression.

**Figures 7–9.**
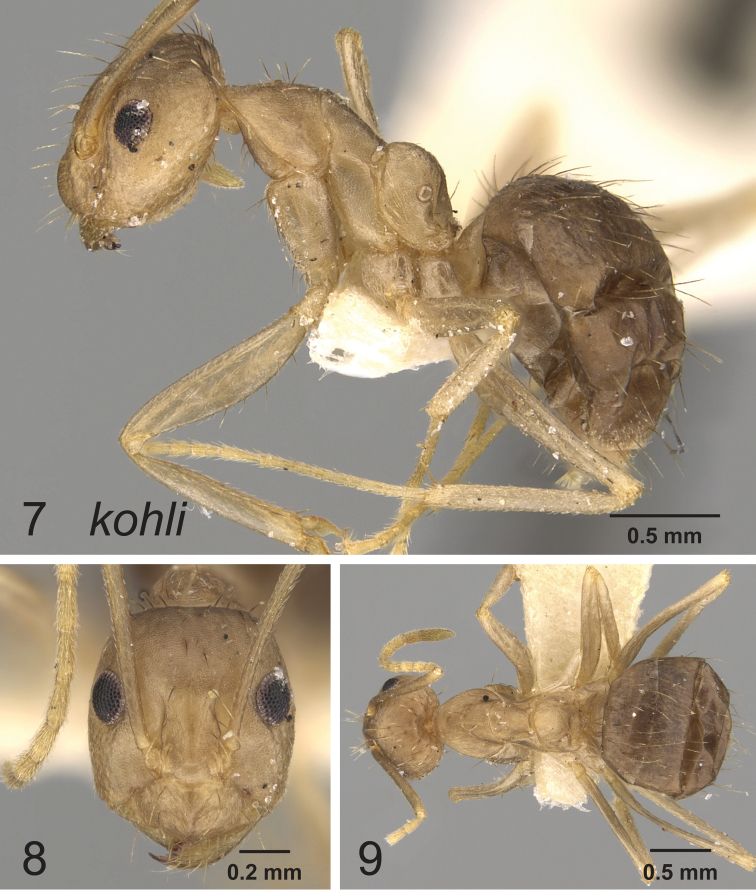
Lateral, full face and dorsal view of body. *Paratrechina kohli* worker CASENT0249635.

### Notes on *Paratrechina* species

Workers of *Paratrechina* are relatively easy to identify to species based on morphology. In addition to the color and sculpture differences listed in the key and descriptions, the two Malagasy species have shorter scapes than *Paratrechina longicornis* and *Paratrechina zanjensis* (SI less than 195) relative to their head widths. The widespread *Paratrechina longicornis* has the lowest profile of any *Paratrechina* with an almost flat pronotum, mesonotum, and propodeum. Scape macrosetae are variable within the genus with both *Paratrechina longicornis* and *Paratrechina kohli* lacking them altogether, but *Paratrechina ankarana*, *Paratrechina antsingy* and *Paratrechina zanjensis* have abundant scape macrosetae. The REL2 is higher in *Paratrechina longicornis* and *Paratrechina zanjensis* than in the other species, typically well into the upper thirties and low forties. For the other three species the REL2 is typically in the upper twenties and low thirties, reflecting an overall wider head relative to eye length.

Only *Paratrechina longicornis* and *Paratrechina zanjensis* workers come closest to resembling one another and these can be easily separated based on the present or absence of scape macrosetae. Workers of all species, except *Paratrechina kohli*, have three small, but distinct, ocelli present. The number of mandibular teeth on *Paratrechina kohli* is particularly interesting. The tooth on the inner mandibular margin is separated from the basal angle tooth by a short diastema. It would not be surprising to occasionally find a *Paratrechina kohli* worker with more than 8 teeth because the single queen specimen known in collections has a tooth that is divided with two sets of cusps, implying more teeth might occasionally be expressed in individuals. In all other *Paratrechina* workers only five mandibular teeth are present. Males are known only from *Paratrechina longicornis* (LaPolla et al. 2013), so it is impossible at this time to discuss general male characteristics for this genus.

## Acknowledgments

We would like to thank Michele Esposito, from CASC, for her support with databasing, imaging processing, proofreading, and her overall support in the lab. Bob Zuparko also contributed to the curation of the material and for arranging loans. The fieldwork on which this study is based could not have been completed without the gracious support of the Malagasy Arthropod Inventory Team (Balsama Rajemison, Jean-Claude Rakotonirina, Jean-Jacques Rafanomezantsoa, Chrislain Ranaivo, Hanitriniana Rasoazanamavo, Nicole Rasoamanana, Clavier Randrianandrasana, Njaka Ravelomanana, and Manoa Ramamonjisoa). Two anonymous reviewers provided helpful and useful comments on the manuscript. This study was supported in part by the National Science Foundation under grants DEB-0743542 to JSL and DEB-0842395 and DEB-0072713 to BLF.

## Supplementary Material

XML Treatment for Paratrechina ankarana

XML Treatment for Paratrechina antsingy

XML Treatment for Paratrechina kohli
